# Early Genomic Detection of Cosmopolitan Genotype of Dengue Virus Serotype 2, Angola, 2018

**DOI:** 10.3201/eid2504.180958

**Published:** 2019-04

**Authors:** Sarah C. Hill, Jocelyne Neto de Vasconcelos, Bernardo Gutierrez Granja, Julien Thézé, Domingos Jandondo, Zoraima Neto, Marinela Mirandela, Cruz dos Santos Sebastião, Ana Luísa Micolo Cândido, Carina Clemente, Sara Pereira da Silva, Túlio de Oliveira, Oliver G. Pybus, Nuno R. Faria, Joana Morais Afonso

**Affiliations:** University of Oxford, Oxford, United Kingdom (S.C. Hill, B. Gutierrez Granja, J. Thézé, O.G. Pybus, N.R. Faria);; Instituto Nacional de Investigação em Saúde, Luanda, Angola (J. Neto de Vasconcelos, D. Jandondo, Z. Neto, M. Mirandela, C. dos Santos Sebastião, A.L.M. Cândido, J.M. Afonso);; Universidad San Francisco de Quito, Colegio de Ciencias Biológicas y Ambientales, Quito, Ecuador (B. Gutierrez Granja);; Cligest, Luanda (C. Clemente, S.P. da Silva); University of KwaZulu-Natal, Durban, South Africa (T. de Oliveira)

**Keywords:** dengue, DENV, vector-borne infections, mosquito-borne diseases, genomic detection, nanopore sequencing, epidemiologic surveillance, viruses, arboviruses, Angola, dengue virus

## Abstract

We used portable genome sequencing to investigate reported dengue virus transmission in Angola. Our results show that autochthonous transmission of dengue serotype 2 (cosmopolitan genotype) occurred in January 2018.

In Africa, the prevalence of disease caused by *Aedes* mosquito–borne virus infections might be similar to that in the Americas (*1*,*2*). However, the transmission and genetic diversity of arthropodborne viruses (arboviruses) in Africa remains poorly understood because of a paucity of systematic surveillance. Moreover, syndromic surveillance might confound symptomatically similar illnesses, and serologic diagnostic tests can be misleading because of cross-reactivity between related circulating flaviviruses (*3*). Improved viral genomic surveillance can assist in better understanding viral transmission dynamics in Africa.

During 2013, Angola experienced a large dengue outbreak that was concentrated in Luanda Province (*4*). Cases detected in travelers returning from Angola to other parts of the world showed that the virus rapidly disseminated from Angola to Europe, Asia, and the Americas (*5*). Although infections were predominantly caused by dengue virus (DENV) serotype 1 (*6*), all DENV serotypes were reported in returning travelers from Angola (*7*).

Although dengue is probably endemic in Angola, patterns of DENV transmission in the country outside the 2013 epidemic are poorly characterized. The lack of genomic characterization restricts our understanding of DENV diversity within Angola and the frequency and directionality with which DENVs are exchanged with other countries. Although enhanced viral genomic sequencing capacity can improve outbreak detection and response, acquiring this tool remains challenging for many public health laboratories because of the high startup costs of traditional bench-top sequencing (*8*). Recent technologic advances now permit more affordable sequencing using the MinION portable sequencer (Oxford Nanopore Technologies, https://nanoporetech.com). We used a combination of portable sequencing and genetic analysis to characterize the causative lineage of a DENV outbreak in Luanda.

## The Study

To investigate the timing and frequency of dengue occurrence in Angola, we conducted rapid diagnostic tests by using the SD Bioline Dengue Duo kit (Alere, https://www.alere.com) to detect the presence of dengue-specific IgM, IgG, and, since January 2017, nonstructural protein 1 (NS1) (Appendix Figure). During January 1, 2016–May 15, 2018, we collected samples from 6,839 patients (3,276 male, 3,563 female) in central Luanda for whom a physician suspected dengue as the cause of an illness with symptoms consistent with DENV infection. Samples were originally obtained for routine diagnostic purposes from persons visiting local clinics. Thus, we used residual samples without informed consent, with ethics approval from the National Ethical Committee of the Angola Ministry of Health.

We identified 80 DENV NS1–positive cases among the tested samples (Appendix Figure). The first confirmed infections were detected in May 2017, and the number of cases appeared to peak around May 2018 (Figure, panel A).

**Figure:**
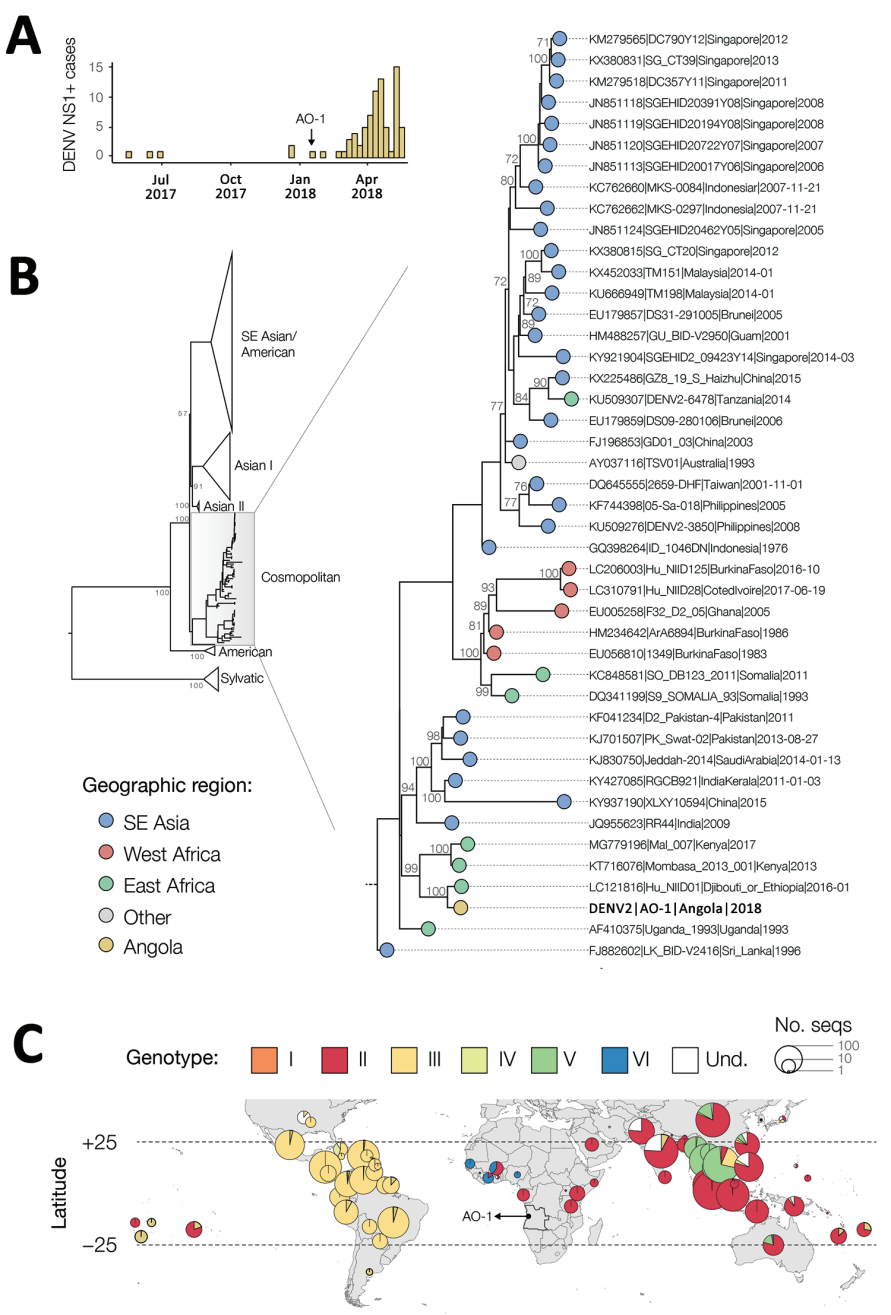
Investigation of DENV infections in Luanda, Angola, January 1, 2016–May 15, 2018. A) Number of DENV infections (i.e., cases positive for DENV NS1), Luanda, Angola, May 1, 2017–May 15, 2018. B) Midpoint rooted maximum-likelihood phylogeny of DENV-2 whole genomes. Support for branching structure is shown by bootstrap values at nodes. On the right side, the cosmopolitan genotype clade containing the Angola DENV-2 sequence is expanded. Colors indicate geographic location of sampling. The Angola DENV-2 is shown in bold. Support for branching structure is shown by bootstrap values at nodes (bootstrap scores >70 shown). C) Geographic distribution of available DENV-2 sequence data (>100 bp). Pie chart radii are log-proportional to the number of sequences available in each country and are colored according to genotype DENV, dengue virus; DENV-2, dengue virus serotype 2; NS1, nonstructural protein 1; SE, Southeast; seq, sequences; und, undefined.

We tested 153 randomly selected serum samples (IgG, IgM, or NS1-positive) collected during July 21, 2017–January 31, 2018, for DENV RNA by using real-time quantitative reverse transcription PCR (qRT-PCR) at the Instituto Nacional de Investigação em Saúde (INIS) in Luanda, Angola. We used the US Centers for Disease Control and Prevention’s DENV1–4 Real-Time RT-PCR assay kit on an Applied Biosystems 7500 Fast Real-Time PCR System (https://www.thermofisher.com), according to the manufacturer’s instructions (*7*). Of these 153 samples, 1 (isolate AO-1) yielded a qRT-PCR cycle threshold of 22.5 for DENV serotype 2 (DENV-2). This sample was obtained from a 48-year-old man living in Luanda who visited a clinic on January 18, 2018. The patient reported traveling to Mussulo Island, a resort 30 km from Luanda, during December 24, 2017–January 2, 2018.

We subjected the qRT-PCR–positive sample to viral genomic amplification and sequencing by using a multiplex PCR primer scheme designed to amplify the entire coding region of DENV-2. We aligned published genomes of nonsylvatic DENV-2 and used them to generate a 90% consensus sequence that formed the target for primer design. We designed primers that generated overlapping amplicons 980 bp in length with an overlap of 20 bp as previously described (*9*; Appendix Table). Details of cDNA synthesis, multiplex PCRs, library preparation, sequencing, and generation of consensus sequences are described in the Appendix.

We originally used the 90% consensus sequence as a reference genome for mapping sequencing reads, but we later refined this reference to a more appropriate reference genome (GenBank accession no. LC121816) by using BLAST (http://www.ncbi.nlm.nih.gov/BLAST) to identify DENV sequences with high identity to the provisionally mapped data. The median sequencing depth was 11,448 reads, and 75% of the genome had a depth of at least 2,419 reads. In total, we sequenced 96% of the coding region of DENV-2 (isolate AO-1; GenBank accession no. MH460898). Raw and processed data are available on GitHub (Appendix).

We constructed phylogenetic trees to explore the relationship of the sequenced AO-1 genome to those of other isolates. We retrieved 1,395 DENV-2 genome sequences with associated date and country of collection from GenBank. From this dataset, we generated a subset that included all 35 identified sequences from Africa, 200 globally sampled sequences (randomly sampled from the remaining 1,360 sequences), and the novel AO-1 sequence. We aligned these sequences by using MUSCLE, as implemented in Geneious 9.0.5 (*10*). We constructed a maximum-likelihood phylogenetic tree by using a general time-reversible model with gamma distributed among site rate heterogeneity (4 categories) in RAXML version 8.2.10 (*11*). We performed 500 nonparametric bootstrap replicates to evaluate statistical support for phylogenetic nodes. We also constructed a phylogeny that includes additional partial gene sequences (Appendix).

Phylogenetic estimation strongly supports placement of the isolate from Angola in the cosmopolitan genotype of DENV-2 (Figure, panel B). The Angola strain forms part of a well-supported monophyletic clade that comprises genomes sampled in East Africa and is most closely related to DENV isolated from a returning traveler from this region. Viruses from this clade have been present in East Africa since at least 2013 (Figure, panel B).

To explore the global distribution of the DENV-2 cosmopolitan genotype and identify geographic gaps in DENV genomic surveillance that might bias phylogenetic interpretation, we generated maps of the distribution of currently available DENV sequence data. We downloaded from GenBank all DENV sequences >100 bp of any serotype with known location of sampling (including those from returning travelers). We genotyped DENV-2 sequences by using the Genome Detective online classification tool (http://www.genomedetective.com). Most sequenced DENVs in Africa belong to DENV-2 (49%), of which 70% belong to the cosmopolitan genotype (Figure, panel C). We found that although 16% of all global clinically apparent dengue infections have been estimated to occur in Africa (*2*), DENV serotype 1–4 sequences from Africa currently represent <1% of the available global DENV sequence data. No data exist from the Democratic Republic of the Congo, which has been epidemiologically linked with Angola during past arbovirus outbreaks (*12*). Additional data will help to address transmission dynamics of DENV-2 in Angola and identify common routes of virus importation into the country.

## Conclusions

The DENV-2 portable sequencing approach we describe represents a useful tool for genomic characterization and molecular epidemiology of outbreaks in Africa and elsewhere. On the basis of phylogenetic evidence and the geographic distribution of detected genotypes, the DENV-2 cosmopolitan genotype detected in Angola is probably endemic in Africa. The AO-1 genome we analyzed probably represents an early transmission event from an ongoing DENV-2 epidemic in Luanda. Further sequencing of DENV in the region is required to determine whether the cosmopolitan genotype is endemic to Angola or if it represents a more recent introduction from elsewhere (e.g., East Africa or other unsampled locations).

Appendix Additional information regarding early genomic detection of cosmopolitan genotype of dengue virus serotype 2, Angola, 2018

## References

[1] Gubler DJ. Dengue and dengue hemorrhagic fever. Clin Microbiol Rev. 1998;11:480–96. 10.1128/CMR.11.3.480 9665979 PMC88892

[2] Bhatt S, Gething PW, Brady OJ, Messina JP, Farlow AW, Moyes CL, et al. The global distribution and burden of dengue. Nature. 2013;496:504–7. 10.1038/nature12060 23563266 PMC3651993

[3] Rabe IB, Staples JE, Villanueva J, Hummel KB, Johnson JA, Rose L, et al.; MTS. Interim guidance for interpretation of Zika virus antibody test results. MMWR Morb Mortal Wkly Rep. 2016;65:543–6. 10.15585/mmwr.mm6521e1 27254248

[4] Centers for Disease Control and Prevention (CDC). Ongoing dengue epidemic - Angola, June 2013. MMWR Morb Mortal Wkly Rep. 2013;62:504–7. 23784016 PMC4604895

[5] Schwartz E, Meltzer E, Mendelson M, Tooke A, Steiner F, Gautret P, et al. Detection on four continents of dengue fever cases related to an ongoing outbreak in Luanda, Angola, March to May 2013. Euro Surveill. 2013;18:20488. 23725977

[6] Parreira R, Centeno-Lima S, Lopes A, Portugal-Calisto D, Constantino A, Nina J. Dengue virus serotype 4 and chikungunya virus coinfection in a traveller returning from Luanda, Angola, January 2014. Euro Surveill. 2014;19:20730. 10.2807/1560-7917.ES2014.19.10.20730 24650864

[7] Abreu C, Silva-Pinto A, Lazzara D, Sobrinho-Simões J, Guimarães JT, Sarmento A. Imported dengue from 2013 Angola outbreak: Not just serotype 1 was detected. J Clin Virol. 2016;79:77–9. 10.1016/j.jcv.2016.04.011 27107210

[8] Gardy JL, Loman NJ. Towards a genomics-informed, real-time, global pathogen surveillance system. Nat Rev Genet. 2018;19:9–20. 10.1038/nrg.2017.88 29129921 PMC7097748

[9] Quick J, Grubaugh ND, Pullan ST, Claro IM, Smith AD, Gangavarapu K, et al. Multiplex PCR method for MinION and Illumina sequencing of Zika and other virus genomes directly from clinical samples. Nat Protoc. 2017;12:1261–76. 10.1038/nprot.2017.066 28538739 PMC5902022

[10] Kearse M, Moir R, Wilson A, Stones-Havas S, Cheung M, Sturrock S, et al. Geneious Basic: an integrated and extendable desktop software platform for the organization and analysis of sequence data. Bioinformatics. 2012;28:1647–9. 10.1093/bioinformatics/bts199 22543367 PMC3371832

[11] Stamatakis A. RAxML version 8: a tool for phylogenetic analysis and post-analysis of large phylogenies. Bioinformatics. 2014;30:1312–3. 10.1093/bioinformatics/btu033 24451623 PMC3998144

[12] Kraemer MUG, Faria NR, Reiner RC Jr, Golding N, Nikolay B, Stasse S, et al. Spread of yellow fever virus outbreak in Angola and the Democratic Republic of the Congo 2015-16: a modelling study. Lancet Infect Dis. 2017;17:330–8. 10.1016/S1473-3099(16)30513-8 28017559 PMC5332542

