# Supplementary material for: Early Genomic Detection of Cosmopolitan Genotype of Dengue Virus Serotype 2, Angola, 2018
Source: Emerg Infect Dis. 2019 Apr;25(4):784–7. doi: 10.3201/eid2504.180958 (PMC6433006; doi:10.3201/eid2504.180958)
Supplement: Appendix — Additional information regarding early genomic detection of cosmopolitan genotype of dengue virus serotype 2, Angola, 2018 [file 18-0958-Techapp-s1.pdf]

Article DOI: https://doi.org/10.3201/eid2504.180958

Early Genomic Detection of Cosmopolitan Genotype of Dengue Virus Serotype 2, Angola, 2018

Appendix

DENV2 Complete Genome MinION Nanopore Sequencing

Between the 15 and 23rd February 2018, we attempted sequencing using the Oxford Nanopore MinION device at Instituto Nacional Investigação em Saúde (INIS), Ministry of Health of Angola, Luanda, on the AO-1 isolate, as part of the ArboSPREAD project focused on genomic surveillance of arthropod-borne viruses. Diagnostic, sequencing and genetic analysis results were presented at the INIS to local public health authorities on the 23rd February 2018. The detailed sequencing protocol has been previously described in (*1*). Following cDNA synthesis using random primers, multiplex PCR is conducted to generate overlapping amplicons of the whole genome of the targeted viral strain (*1*). Extracted RNA was reverse-transcribed to cDNA using the Protoscript II First Strand cDNA synthesis Kit (New England Biolabs, Hitchin, UK) and random hexamer priming. DENV2 genome amplification was attempted using 35 cycles of PCR according to the reaction mix and thermocycling conditions given in Quick et al (*1*), and with the primers shown in Appendix Table. PCR products were cleaned up using AmpureXP purification beads (Beckman Coulter, High Wycombe, UK) and quantified with the Qubit dsDNA High Sensitivity assay on a Qubit 3.0 instrument (Life Technologies). Presence of correctly-sized bands were checked on an E-Gel electrophoresis machine. PCR products for the AO-1 sample were barcoded using the Native Barcoding Kit (NBD103, Oxford Nanopore Technologies, Oxford, UK) and pooled in an equimolar fashion. Sequencing libraries were generated from the barcoded products using the Genomic DNA Sequencing Kit SQK-LSK108 (Oxford Nanopore Technologies). We used 250 ng of total DNA input in the library preparation. The library was loaded onto a flow cell (FLO-MIN106) and sequencing data were collected for 24 hours.

Processing of Sequencing Data and Generation of Consensus Sequences

Consensus sequences were generated from raw data reads using a pipeline that has been extensively described previously (*1*), with only minor modifications. First, raw data were basecalled using Albacore Sequencing Pipeline Software version 2.1.10 (Oxford Nanopotre Technologies). Basecalled data were demultiplexed into separate barcodes using Porechop version 0.2.3 (https://github.com/rrwick/Porechop), with reads being assigned to barcodes only when barcodes were present at both ends of the sequence. Demultiplexed data were subject to consensus calling using python scripts (the main script 'zibra.py' and all other scripts called by this code are available at https://github.com/zibraproject/zika-pipeline, and have been previously detailed in the scientific literature [*1*]). Briefly, each basecalled FASTA file is mapped to the reference genome using bwa v 0.7.16a-r1181 (http://bio-bwa.sourceforge.net/bwa.shtml#13) and a BAM file is produced. Primers are trimmed based on positions relative to the reference genome given in input BED files and coverage is normalized to improve computational speed. Nanopolish (version 0.8.4) (https://github.com/jts/nanopolish) is used to call variants. Variants with $\geq$20 depth are used to generate consensus sequences, and regions with lower coverage, and those in primer binding regions were masked with N characters. To prevent our consensus sequence being affected by the reference sequence chosen, we first mapped basecalled reads to a 90% consensus sequence of a representative non-sylvatic DENV2 alignment and extracted the consensus of this. The generated consensus was then used as input to a BLAST query to find the closest identity genome available in GenBank. The closest identity sequence (GenBank accession number: LC121816) was then reused as the reference genome and the consensus generation pipeline was rerun. Key data files produced by the pipeline, input BED and reference files, and raw FAST5 files linked to the barcodes associated with dengue that were included in this library are available on GitHub (https://github.com/arbospread/DENV2-Angola-2018_01).

Partial Gene Phylogeny

Most DENV sequences available in GenBank are partial gene sequences. We therefore supplemented the whole genome alignment with all DENV2 sequences >1000 bp from Africa that belonged to the same genotype as the novel sequence from Angola. Sequences were aligned to the envelope region of DENV2, and we estimated a separate phylogeny from this alignment

by using the models we have described. The closest relative to AO-1 was not affected by inclusion of these partial sequences, and no additional diversity (i.e., DENV sampled from other years and/or countries) was included within the monophyletic African clade containing AO-1, so this tree is not shown.

Reference

1. Quick J, Grubaugh ND, Pullan ST, Claro IM, Smith AD, Gangavarapu K, et al. Multiplex PCR method for MinION and Illumina sequencing of Zika and other virus genomes directly from clinical samples. Nat Protoc. 2017;12:1261–76. http://dx.doi.org/10.1038/nprot.2017.066 PubMed

Appendix Table. Sequencing primers used for MinION sequencing of DENV2.

Primer Name	Primer sequence (5′-3′)
DENV2_1_LEFT	AGCAGATCTCTGATGAATAACCAACG
DENV2_1_RIGHT	TTTTTGCCATCGTCGTCACACA
DENV2_2_LEFT	TCGCTCCTTCAATGACAATGCG
DENV2_2_RIGHT	CCATTCTCAGCCTGCACTTGAG
DENV2_3_LEFT	ACATTGGTCACTTTCAAAAATCCCC
DENV2_3_RIGHT	TGAAGGGGATTCTGGTTGGAACT
DENV2_4_LEFT	ATAGTGGTTGCGTTGTGAGCTG
DENV2_4_RIGHT	CGGCAGCACCATTCTGTTATGA
DENV2_5_LEFT	TCATGCAGGCAGGAAAACGATC
DENV2_5_RIGHT	TCTCAAGAGTAGTCCAGCTGCA
DENV2_6_LEFT	TGGAAATCAGACCATTGAAAGAGAAAGA
DENV2_6_RIGHT	TGGTCAGTGTTTGTTCTTCCTCTT
DENV2_7_LEFT	CCAATCCTGTCAATAACAATATCAGAAGAT
DENV2_7_RIGHT	TGATGGCTGGGGTTTGGTATCT
DENV2_8_LEFT	AGATCGAAGATGACATTTTCCGAAAGA
DENV2_8_RIGHT	CCCATGTATATGTACTGGTCATTTTCATT
DENV2_9_LEFT	ATGCCAGTGACCCACTCTAGTG
DENV2_9_RIGHT	CCACCACTGTGAGGATGGCTAT
DENV2_10_LEFT	ACCAGAAAAACAGAGAACACCCC
DENV2_10_RIGHT	CCACTTCCTGGATTCCACTTTTCT
DENV2_11_LEFT	GGAGCTGGACTTCTCTTTTCCAT
DENV2_11_RIGHT	GACGTCCCAAGGTTTTGTCAGC
DENV2_12_LEFT	AGAGCATGAAACATCATGGCACT
DENV2_12_RIGHT	GTGCCTCTTGGTGTTGGTCTTT
DENV2_13_LEFT	TGGGACACAAGAATCACACTAGAAG
DENV2_13_RIGHT	CCGCACCATTGGTCTTCTCTTT

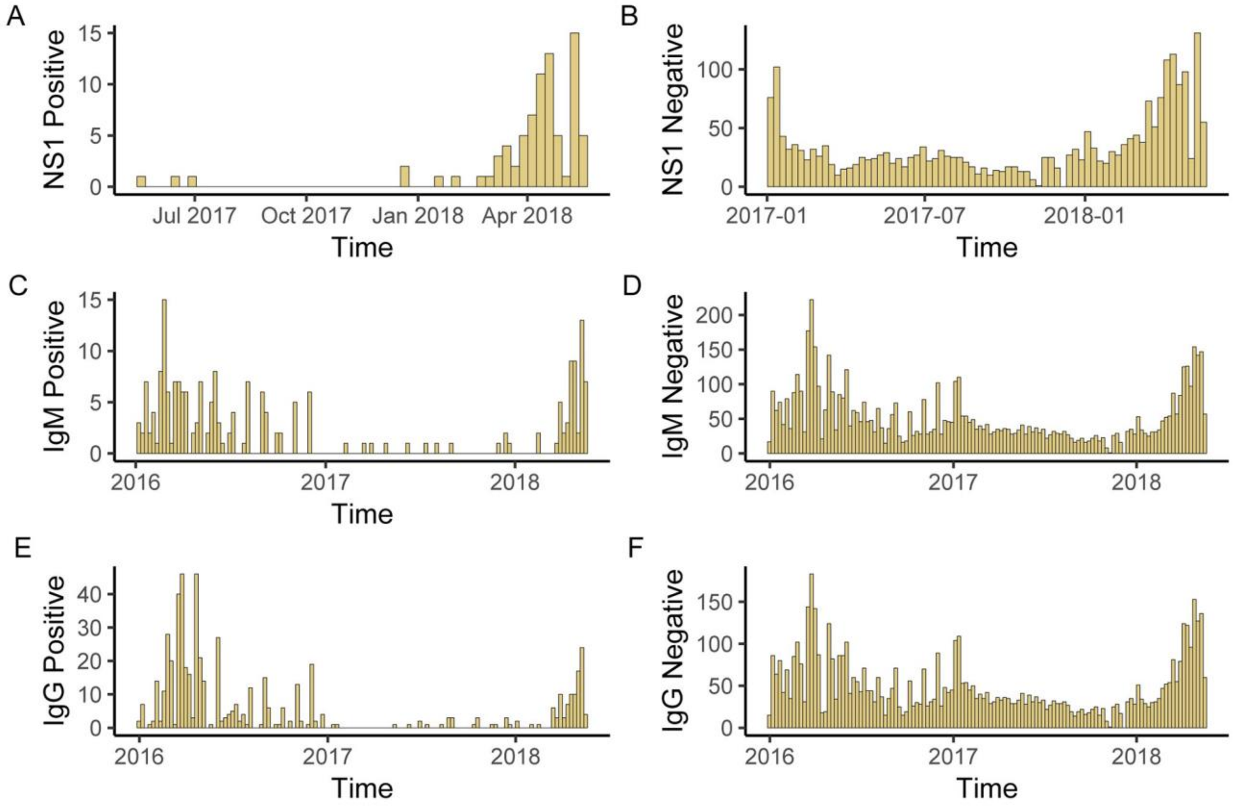

Appendix Figure. Summary of the serologic results obtained from 6,839 tests performed in Luanda between Jan 2016 and 15th May 2018. Panels A, C and E show positive results; panels B, D and F indicate number of negative results through time. Note that while IgM (panels C and D) and IgG (panels E and F) screening started in Jan 2016, NS1 screening (panels A and B) started only in Jan 2017. The IgM and IgG positive cases throughout 2016 are possibly due to the presence of antibodies against yellow fever virus.
